# A physiologic flow phantom for the evaluation of 4D flow MRI in the left ventricle

**DOI:** 10.1186/1532-429X-17-S1-Q106

**Published:** 2015-02-03

**Authors:** Ikechukwu (Ikay) Okafor, Christine Garcia, Alex J Barker, John Oshinski, Ajit P Yoganathan

**Affiliations:** 1 Biomedical Engineering, Georgia Institute of Technology, Atlanta, GA, USA; 2 Radiology, Emory University, Atlanta, GA, USA; 3 Biomedical Engineering, Emory University, Atlanta, GA, USA; 4 Radiology, Northwestern University, Chicago, IL, USA

## Background

4D PCMR has the potential to help in understanding the direct relationship between fluid and structure interaction within the cardiovascular system, to allow for 3D visualization of blood flow patterns in the heart, and to make direct wall shear stress measurements. However, the wide dynamic range of velocities in the left ventricle (LV, range of 0-200 cm/s) can create difficulties when optimizing acquisition protocols, especially when attempting to measure complex flow features during filling and diastasis. Additionally, there is no reference standard method to validate velocity flow and wall shear stress measurements. Here we describe our experience with a custom-built MRI compatible physiologic left heart flow phantom which can be used to optimize acquisition parameters, and can be used in the lab to make velocity measurements with particle image velocimetry (PIV).

## Methods

**Figure 1:**
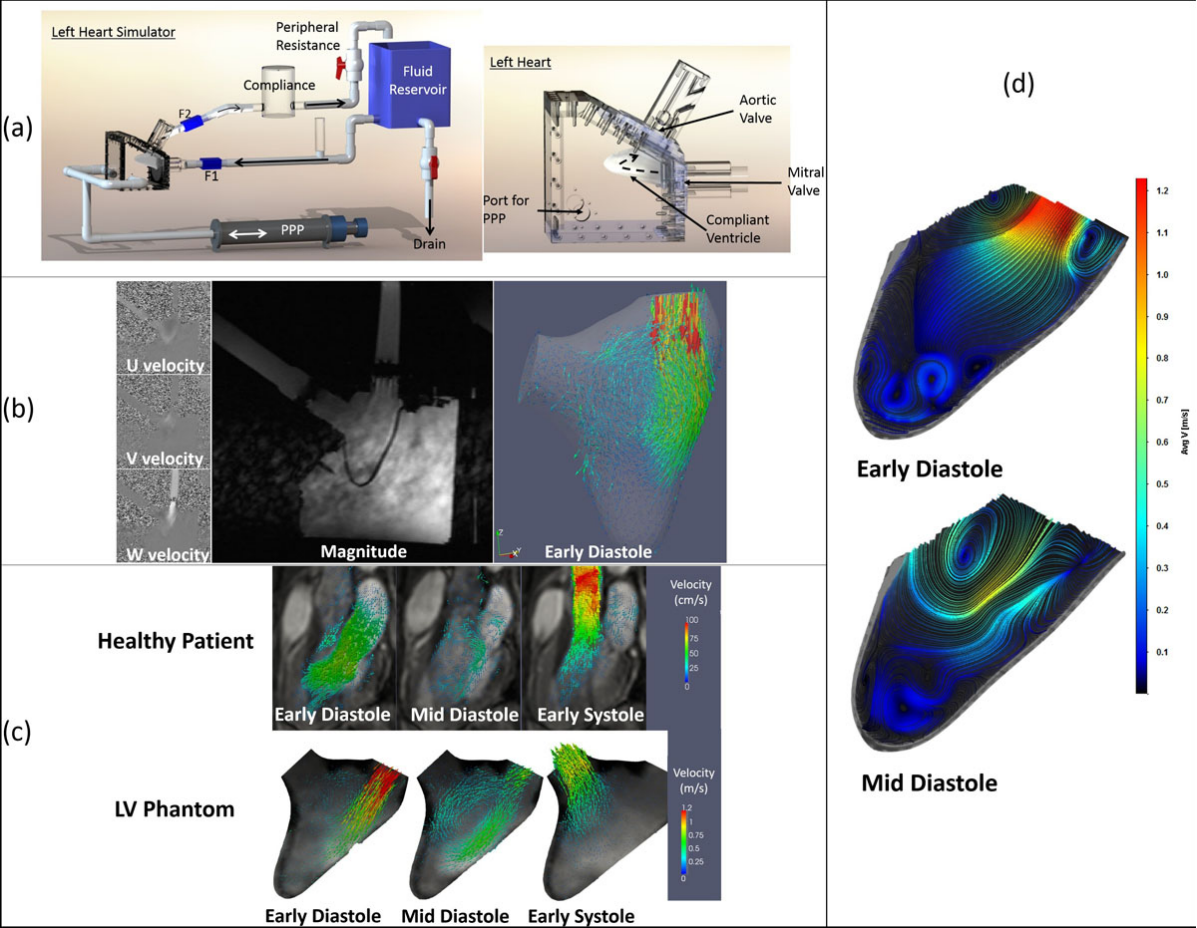
Schematic of the physiologic flow phantom simulator consisting of adjustable compliance and resistance. F1 and F2 are flow probes which can be used to measure flow rates into and out of the left ventricle (Right). Left heart. PPP stands for programmable piston pump (Left). (b): Sample raw images from 4D sequence (Left) and extracted ventricular wall and 3D velocity vectors at the beginning of diastole (Right). (c) Comparison of LVOT slice between the LV flow phantom and a healthy volunteer at 3 time points in the cardiac cycle.(d) PIV of diastolic flow within the LV

The left heart flow phantom (Figure 1a) consists of a left ventricular (LV) geometry based on cine MRI scans of a healthy patient. The contraction and relaxation of the LV is controlled via a linearly actuated piston pump. Valves are placed in the mitral and aortic position to generate physiological flow conditions. Adjustable systemic resistances and compliances are used to tune the system to attain physiologic cardiac output and pressures. A 4D PCMR scan was performed in a Siemens Trio 3.0T magnet with the image volume adjusted to cover the entire LV. The scan parameters for the pilot scan are summarized in Table 1. Cardiac gating was used via TTL triggering, the heart rate was programmed to 70 bpm. The images were post-processed and visualized using in-house code and Paraview.

**Table 1:** 4D Scan Parameters

Acquisition Parameter	Setting
Echo Time	3.7650 ms

Repetition Time	26.4 ms

Flip Angle	15°

Velocity Encoding	150 cm/s

Spatial Resolution	1.172 × 1.172 × 3.79 mm

# Slices	20

Temporal Resolution	29 phases/cycle (33ms)

Total Scan Time	~ 12 minutes

## Results

The 4D PCMR images captured the 3D velocity field within the LV throughout the cardiac cycle. The spatial and temporal resolution chosen was sufficient enough to extract and reconstruct the temporal motion of the ventricular wall (Figure 1b). The analysis of the extracted 3D velocity field showed and inflow peak velocity of 1.2 m/s through the mitral valve and an outflow of 1 m/s through the aortic valve. Left ventricular outflow tract (LVOT) flow in the phantom showed good qualitative (large flow structures) and quantitative (peak velocities for in and outflow) agreement with PCMR images of a healthy volunteer as well as the PIV data (Figure 1c and d).

## Conclusions

A 4D PCMR scan was performed on the left heart flow phantom developed for this study. The scan parameters used here found that 3D blood flow and wall motion information were extracted; nonetheless, further optimization such as k-t undersampling may allow for higher resolution and shorter scan times. The left heart flow phantom can be used to identify (or optimize) alternative MRI sequences to research valvular and ventricular pathologies.

## Funding

NHLBI (RO1HL07262).

